# Knee extensor loss and proximal tibial soft tissue defect managed successfully with simultaneous medial gastrocnemius flap, saphenous fasciocutaneous flap and medial hemisoleus flap: a case report

**DOI:** 10.1186/1752-1947-7-76

**Published:** 2013-03-18

**Authors:** Vignesh P Krishnamoorthy, Dan B Inja, Alfred C Roy

**Affiliations:** 1 Department of Orthopaedics-Unit II, Christian Medical College, Vellore, Tamil Nadu, 632004, India; 2 Department of Orthopaedics-Unit I, Christian Medical College, Vellore, Tamil Nadu, 632004, India

**Keywords:** Knee, Extensor, Gastrocnemius, Saphenous, Hemisoleus

## Abstract

**Introduction:**

Open fractures of the proximal tibia often pose serious challenges to the treating orthopedic surgeon. Management of these complex injuries becomes difficult if they are associated with damage to the extensor mechanism and an exposed knee joint. The scenario becomes more difficult to manage when the soft tissue defect extends to the middle third of the leg. We report a case where we used an extended medial gastrocnemius flap in combination with a saphenous artery fasciocutaneous flap and a medial hemisoleus flap for treatment of an infected proximal tibia fracture with loss of the extensor mechanism and soft tissue defect. To the best of our knowledge, combined use of these three flaps for the management of such injuries has not been reported elsewhere to date.

**Case presentation:**

A 28-year-old Indian man presented to our Out-patient Department with complaints of pain and pus discharge from his left proximal leg for four weeks. He had sustained an open fracture of his left proximal tibia in a road traffic accident five weeks ago and had been operated on elsewhere. He had a stiff, painful knee with an infected wound of 4×4cm over the proximal third of his leg exposing infected, necrotic patellar tendon. He was successfully managed with debridement and simultaneously elevated flaps as described.

**Conclusions:**

This procedure avoids the donor site morbidity associated with free flaps harvested from sites distant from the site of injury, and also does not need the expertise of microvascular reconstruction. To the best of our knowledge, this is also the first report of the combined use of three local flaps for knee extensor reconstruction and soft tissue coverage around the knee.

## Introduction

Open fractures of the proximal tibia often pose serious challenges to the treating orthopedic surgeon because of multiple issues such as the high velocity of the injury causing extensive soft tissue disruption, the intra-articular nature of the fracture and significant metaphyseal comminution with bone loss. Management of these complex injuries becomes more difficult if they are associated with damage to the extensor mechanism and an exposed knee joint. Fracture stabilization, reconstruction of the quadriceps mechanism and coverage of the knee joint and the soft tissue defect involving the proximal leg must be considered simultaneously for faster rehabilitation. There are limited treatment options, particularly when the soft tissue defect extends to the middle third of the leg.

We present a novel method of using three flaps: an extended medial gastrocnemius muscle flap including a part of its distal tendon (hemi-Achilles tendon) for reconstruction of the quadriceps mechanism combined with a saphenous artery fasciocutaneous flap for the additional soft tissue coverage around the proximal tibia. We also used the medial hemisoleus flap combined with these two flaps to cover the soft tissue defect extending to the middle third of the leg.

## Case presentation

A 28-year-old Indian man presented to our Out-patient Department with complaints of pain and pus discharge from his left proximal leg for four weeks. He was unable to bear weight on his left leg and had a stiff left knee. He had sustained a left frontal extra-dural hemorrhage with depressed frontal bone fracture and an open fracture of his left proximal tibia in a road traffic accident five weeks ago. He was treated elsewhere with excision of the depressed skull bone fragment and evacuation of the extra-dural hematoma. Open reduction and internal fixation of the proximal tibia fracture with a medial locking compression plate (LCP®; stainless steel) was performed on the same day using a ‘Mercedes-Benz’ incision. Post-operatively, he was given a knee brace. Our patient developed pus discharge from the surgical wound in the proximal third of his leg three to four days after surgery. As the infection could not be controlled with antibiotics, he was referred to us for further management.

**Figure 1:**
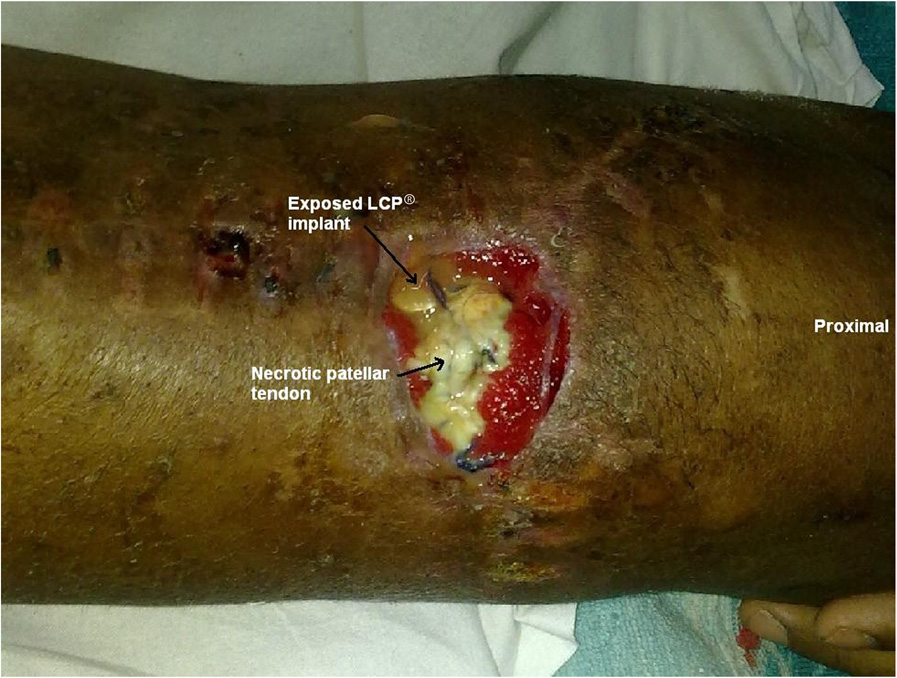
Photograph showing Y-shaped scar over the left knee with raw area exposing necrotic patellar tendon and proximal medial locking compression plate implant.

On examination, there was a Y-shaped scar over the left knee reaching up to the middle third of the leg with three sinuses on the suture line. There was an infected wound of size 4×4cm over the proximal third of the leg exposing necrotic, infected patellar tendon. The medial LCP® implant was also exposed (Figure 1). The left knee was mildly subluxed posteriorly and he had a 10° fixed flexion deformity of his left knee with minimal further flexion. He also had an equinus contracture of his left ankle. A pus sample was sent for culture and sensitivity testing and it showed heavy growth of *Pseudomonas*, sensitive only to imipenem and meropenem. We decided to perform a single stage debridement, with removal of the exposed LCP® implant, reconstruction of the extensor mechanism and fracture stabilization with a knee-spanning external fixator. He was counseled about the need for surgery and the proposed treatment plan and the prognosis was also discussed.

**Figure 2:**
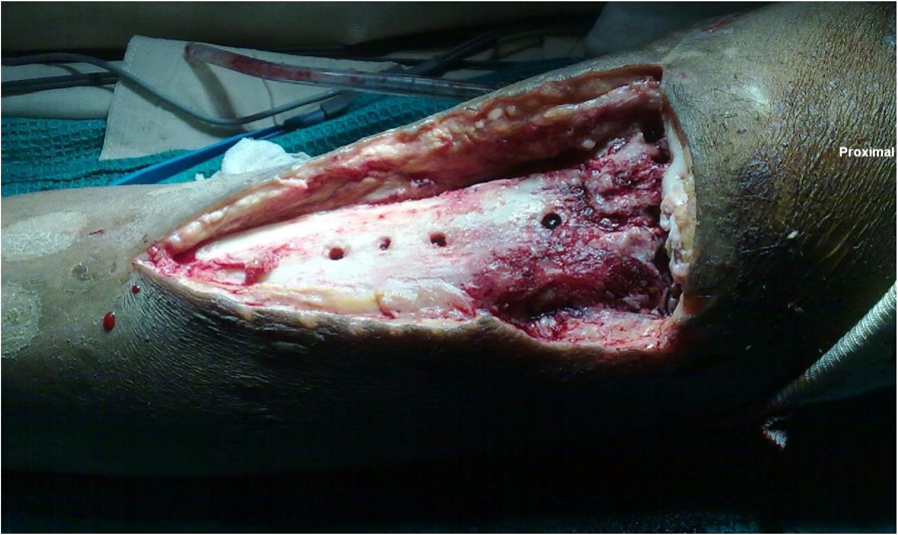
Photograph showing the soft tissue defect over the anterior and medial aspects of the left knee and proximal leg after debridement.

Under combined spinal and epidural anesthesia, our patient was positioned supine with a sandbag under the opposite buttock. The left lower limb was slightly externally rotated and flexed at the knee. The entire left lower limb was prepared and draped, and a sterile tourniquet was used. The right lower limb was also prepared and draped. The old scar over the left knee and the proximal leg including the three sinuses was excised and radical debridement was performed down to the bone. The patellar tendon was exposed and necrotic and hence had to be removed. The proximal medial LCP® implant and screws were removed and the screw holes curetted. The knee joint was also infected with arthrofibrosis and erosions on the articular cartilage. The menisci and the cruciate ligaments were destroyed and hardly identifiable. The proximal tibial fracture had not united and so was fixed with one 16mm partially threaded cancellous screw (6.5×75mm; Synthes GmbH, Switzerland) with a washer. There was a soft tissue defect of 12×7cm over the anterior and medial aspect of the upper third of the tibia with loss of the extensor mechanism of the knee (Figure 2).

**Figure 3:**
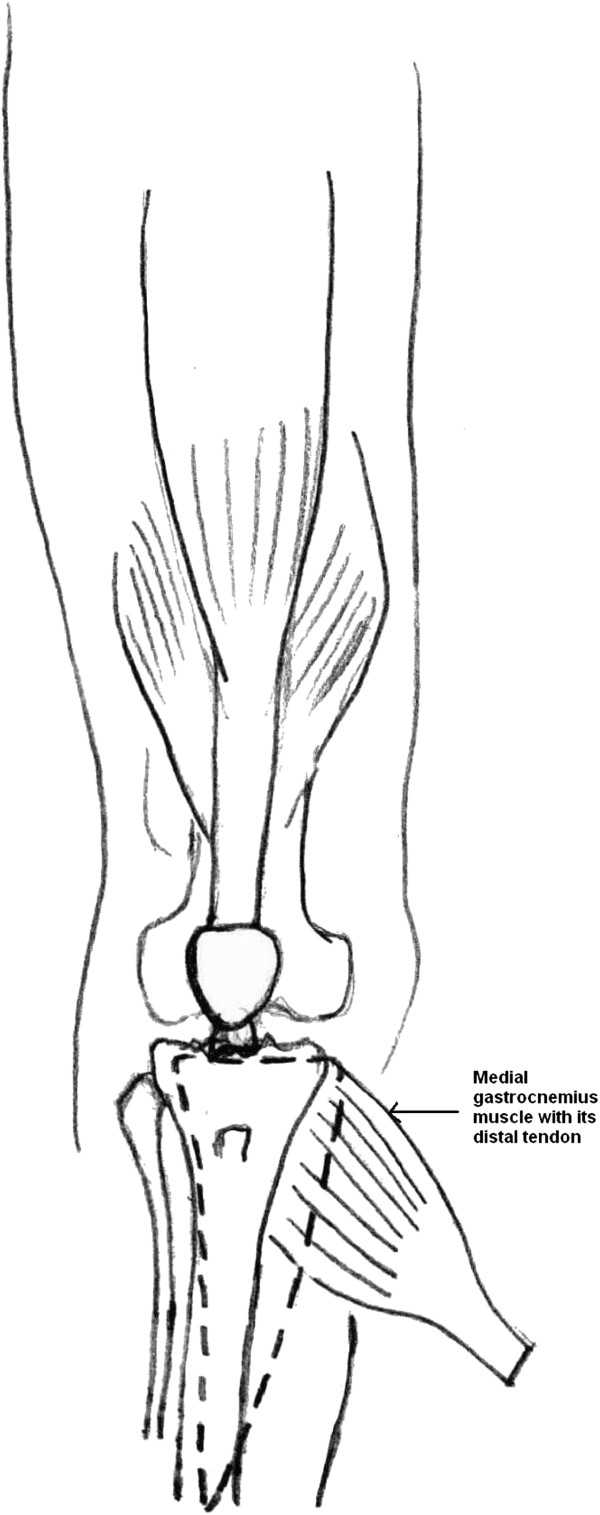
Line diagram illustrating the surgical procedure: illustration shows the medial gastrocnemius brought into the anterior wound under the medial saphenous skin flap.

**Figure 4:**
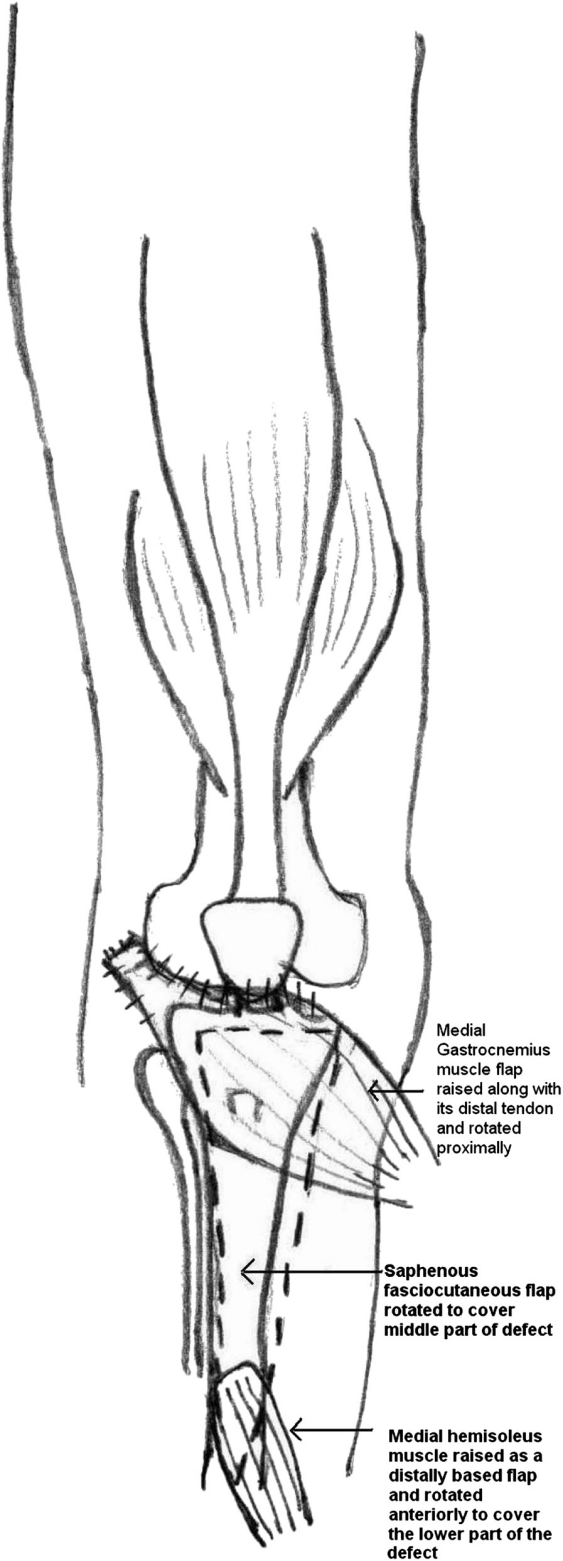
Line diagram showing the medial gastrocnemius and its tendon sutured to remnant patellar tendon, patella and the retinaculum, saphenous fasciocutaneous flap rotated laterally and the medial hemisoleus rotated as a distally-based flap to cover the distal part of the defect.

A 15cm longitudinal incision was made 3cm behind the posteromedial border of the tibia; the medial gastrocnemius muscle was raised as a flap along with its distal tendinous portion (hemi-Achilles tendon) through this incision and was used to cover the antero-medial side of the knee and the upper part of the defect (Figures 3 and 4). The distal hemi-Achilles tendon was sutured to the remnant patellar tendon and the remaining part of the extensor retinaculum with the knee in full extension using Number 1 Vicryl™ (Ethicon, Inc, Somerville, NJ, USA). The bridge of skin between the raw area and the posteromedial skin incision was raised as a proximally-based fasciocutaneous flap, based on the saphenous artery, and was rotated laterally to cover the middle of the defect.

**Figure 5:**
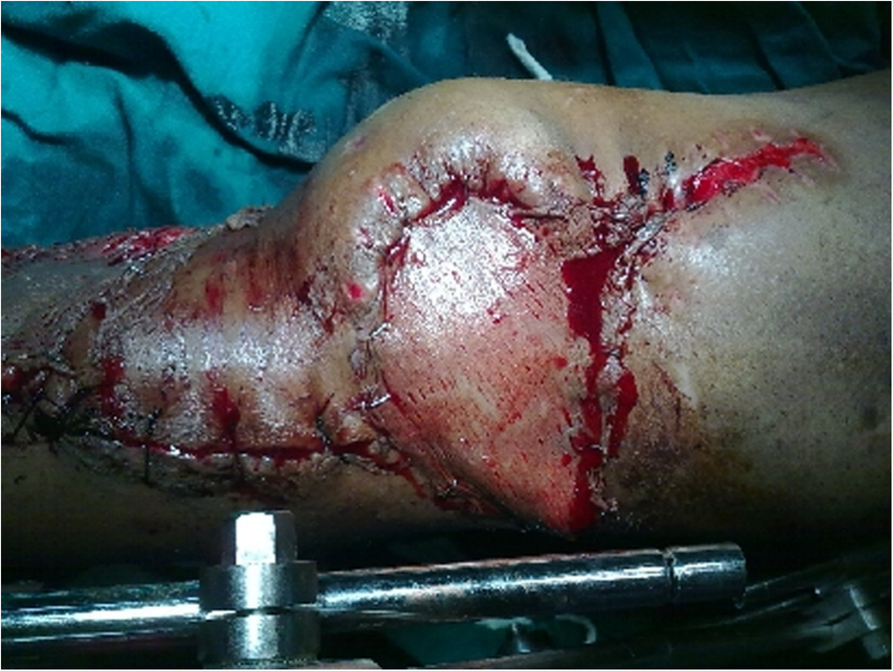
Photograph of left knee and leg showing the medial gastrocnemius flap covered with a split thickness skin graft used for extensor reconstruction, the saphenous fasciocutaneous flap rotated to cover the upper part of defect and the medial hemisoleus covered with a split thickness skin graft in the lower part of the defect.

There was still a residual soft tissue defect of 3×3cm over the junction of the upper third and the middle third of the leg. The posteromedial incision was extended down and the medial hemisoleus was released progressively from the deep flexors and from the lateral hemisoleus at the median raphe. The proximal pedicles from the posterior tibial artery were ligated and divided. The muscle was released as far as the secondary pedicle located at the middle of the leg and was rotated anteriorly as a distally-based flap to cover the residual defect. A split thickness skin graft (STSG) was harvested from the contralateral thigh and was placed over the muscle flaps (Figure 5). The sterile tourniquet was removed and a knee-spanning external fixator applied with three pins each in the tibia and femur. The external fixator was kept in place for four weeks until soft tissue healing had occurred.

**Figure 6:**
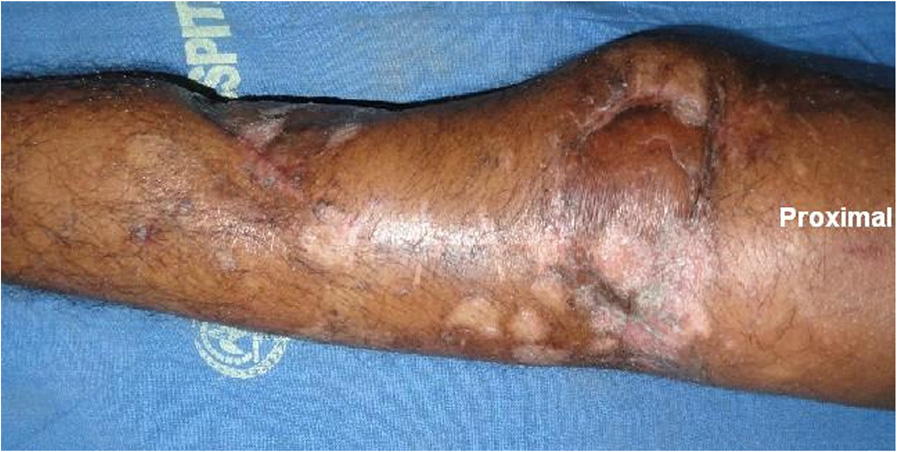
Photograph of our patient’s left leg showing all three flaps healed well with no infection at one year from surgery.

**Figure 7:**
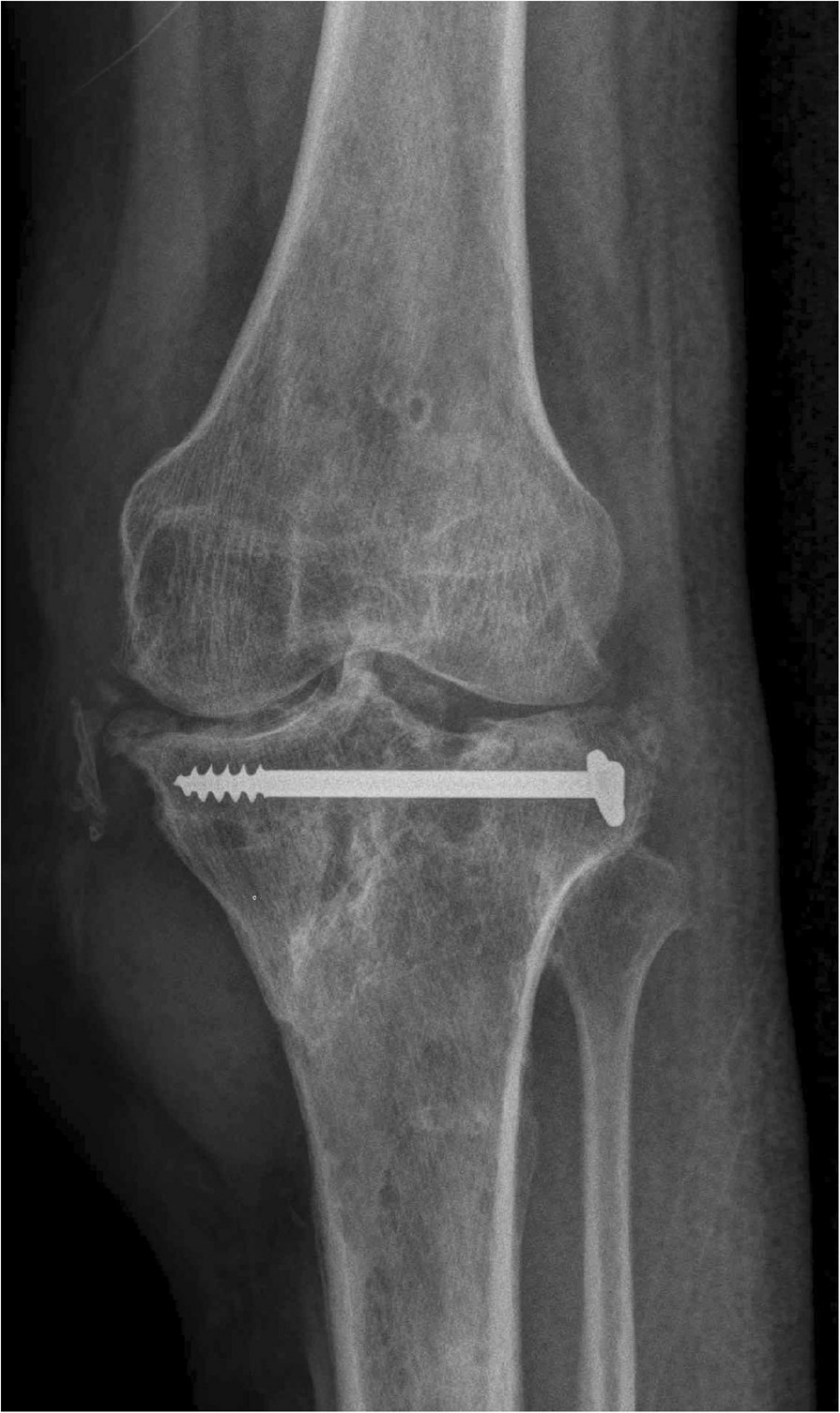
Radiograph (antero-posterior view) of our patient’s left knee and leg, taken one year after surgery, showing the proximal tibia fracture united.

**Figure 8:**
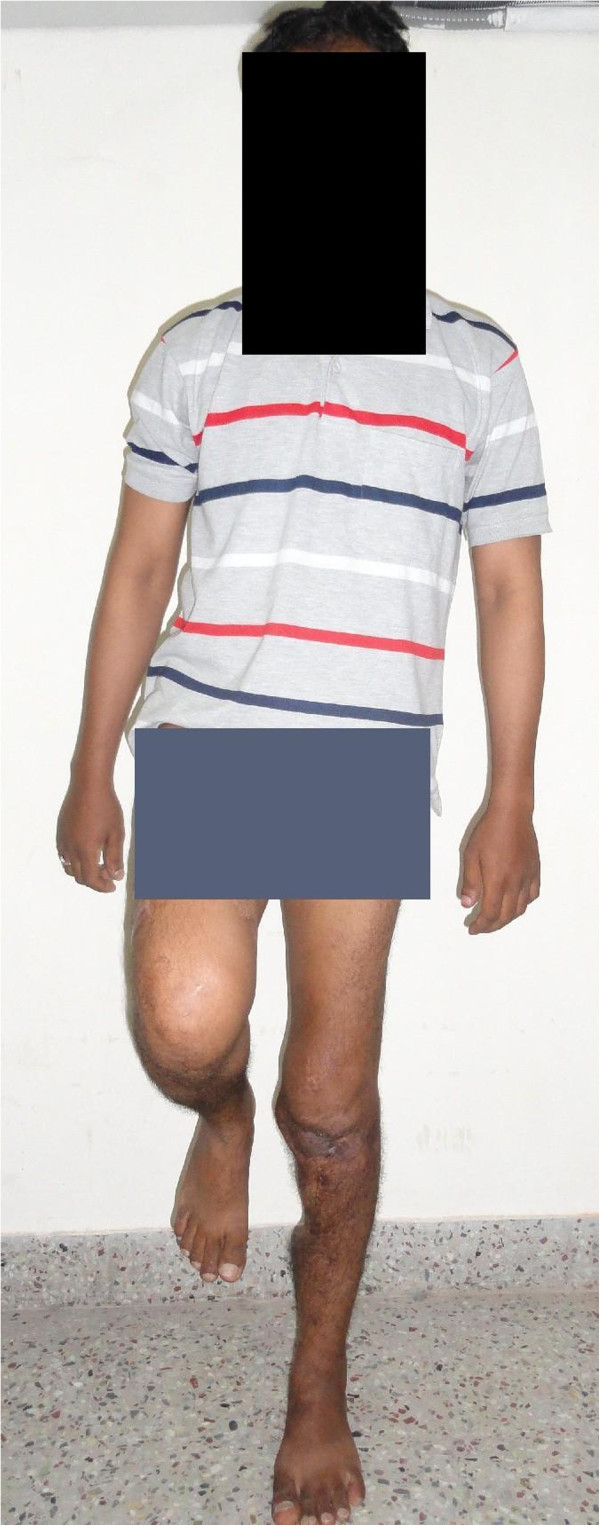
Photograph showing our patient able to stand on the operated left leg alone without support, one year after surgery.

Our patient was started on a quadriceps rehabilitation program and assisted knee range of motion exercises with a hinged knee brace after fixator removal. At one-year follow-up, the flaps had fully healed (Figure 6). The fracture had also completely healed (Figure 7) and our patient was able to walk with full weight-bearing on his left lower limb without pain and without crutches. He was able to stand on the operated leg alone without support (Figure 8). He had an extensor lag of 20° and knee flexion up to 80°. He had some residual posterior subluxation of his left tibia. He may require a knee arthrodesis in the future because of articular cartilage damage caused by pre-existing infection.

## Discussion

High velocity open knee injuries often present a serious challenge for the orthopedic surgeon. They are particularly difficult to manage when associated with disruption of the extensor mechanism of the knee. The nature and severity of open injuries in developing countries are different from those seen in developed countries [1]. In developing countries, patients often present late and the presence of infection adds to the complexity of treatment, often requiring repeated and extensive debridement before reconstruction [2].

There are several options for reconstruction of the quadriceps mechanism including gastrocnemius rotation flap, free latissimus dorsi flap, free rectus femoris flap, semitendinosus autograft and allografts [2]. The gastrocnemius flap has been extensively used for anterior knee cover and reconstruction of extensor mechanism loss of the knee [2]. Jepegnanam *et al*. reported eight cases of traumatic, complete, infected, extensor mechanism loss attributable to high-velocity open knee injuries treated with gastrocnemius flaps [2]. However, the gastrocnemius flap alone does not suffice when the soft tissue defect extends up to the middle third of the leg. In such cases, there are limited treatment options and reconstruction of the quadriceps mechanism and coverage of the soft tissue defect must be considered simultaneously.

Park *et al*. [3] were presented with such a situation after wide debridement for infection after an open reduction and internal fixation of an open, comminuted, patellar fracture. They reported using an extended medial gastrocnemius flap (EMGF) including a tendinous portion of the Achilles for reconstruction of the quadriceps mechanism along with a saphenous neurocutaneous flap (SNCF) for the additional soft tissue coverage around the proximal tibia and patellar tendon [3]. This was the first and only report documenting the use of dual flaps for the treatment of quadriceps mechanism reconstruction and concomitant soft tissue coverage around the knee. To the best of our knowledge there has been no report to date documenting the use of three simultaneously elevated flaps for the management of knee extensor mechanism loss and concomitant soft tissue defect extending up to the middle third of the leg.

We used only the medial gastrocnemius and the medial hemisoleus muscles in our patient’s case and the intact lateral halves of the muscles allow preservation of foot plantar flexion. Also, previous anatomic studies of the soleus muscle clearly demonstrate the bipenniform morphology of the muscle and the independent neurovascular supply to either the medial or lateral belly of the muscle [4]. Therefore, the soleus can be split longitudinally along the median raphe to create a muscle flap composed of only half of the muscle. The most significant advantage of the hemisoleus muscle flap is the preservation of foot plantar flexion power by the other intact muscle belly [5]. Also, the hemisoleus flap has a larger arc of rotation than a conventional soleus muscle flap [4]. The medial part of the muscle is supplied throughout its length by perforators arising from the posterior tibial vessels. This makes the medial hemisoleus reliable as a proximally-based or distally-based flap [6].

## Conclusions

A combination of simultaneously elevated EMGF, a saphenous fasciocutaneous flap and a medial hemisoleus flap can be conveniently used for reconstruction of knee extensor mechanism and for the coverage of the soft tissue defect around the anterior and medial aspects of the knee joint extending down to the middle third of the leg. This procedure does not need the expertise of microvascular reconstruction and also avoids the donor site morbidity associated with free flaps harvested from sites distant from the site of injury.

## Abbreviations

EMGF: extended medial gastrocnemius flap; LCP®: locking compression plate; STSG: split thickness skin graft; SNCF: saphenous neurocutaneous flap.

## Competing interests

The authors declare that they have no competing interests.

## Authors’ contributions

KVP was the lead surgeon who operated on our patient and is the main author of the manuscript. IDB was the assistant surgeon and was a major contributor to writing the manuscript. RAC also contributed to writing the manuscript. All authors read and approved the final manuscript.

## Consent

Written informed consent was obtained from the patient for publication of this case report and any accompanying images. A copy of the written consent is available for review by the Editor-in-Chief of this journal.
